# Challenges of caring for children with mental disorders: Experiences and views of caregivers attending the outpatient clinic at Muhimbili National Hospital, Dar es Salaam - Tanzania

**DOI:** 10.1186/1753-2000-6-16

**Published:** 2012-07-05

**Authors:** Joel Semel Ambikile, Anne Outwater

**Affiliations:** 1 School of Nursing, Muhimbili University of Health and Allied Sciences, P.O Box 65004, Dar es Salaam, Tanzania

**Keywords:** Parents’ challenges, Caregivers challenges, Children, Mental disorders, Mental illness, Africa, Tanzania

## Abstract

**Background:**

It is estimated that world-wide up to 20 % of children suffer from debilitating mental illness. Mental disorders that pose a significant concern include learning disorders, hyperkinetic disorders (ADHD), depression, psychosis, pervasive development disorders, attachment disorders, anxiety disorders, conduct disorder, substance abuse and eating disorders. Living with such children can be very stressful for caregivers in the family. Therefore, determination of challenges of living with these children is important in the process of finding ways to help or support caregivers to provide proper care for their children. The purpose of this study was to explore the psychological and emotional, social, and economic challenges that parents or guardians experience when caring for mentally ill children and what they do to address or deal with them.

**Methodology:**

A qualitative study design using in-depth interviews and focus group discussions was applied. The study was conducted at the psychiatric unit of Muhimbili National Hospital in Tanzania. Two focus groups discussions (FGDs) and 8 in-depth interviews were conducted with caregivers who attended the psychiatric clinic with their children. Data analysis was done using content analysis.

**Results:**

The study revealed psychological and emotional, social, and economic challenges caregivers endure while living with mentally ill children. Psychological and emotional challenges included being stressed by caring tasks and having worries about the present and future life of their children. They had feelings of sadness, and inner pain or bitterness due to the disturbing behaviour of the children. They also experienced some communication problems with their children due to their inability to talk. Social challenges were inadequate social services for their children, stigma, burden of caring task, lack of public awareness of mental illness, lack of social support, and problems with social life. The economic challenges were poverty, child care interfering with various income generating activities in the family, and extra expenses associated with the child’s illness.

**Conclusion:**

Caregivers of mentally ill children experience various psychological and emotional, social, and economic challenges. Professional assistance, public awareness of mental illnesses in children, social support by the government, private sector, and non-governmental organizations (NGOs) are important in addressing these challenges.

## Background

The onset and chronic presence of mental illness in the family can be a stressful event or a crisis for family members [1]. Moving from a crisis to recovery in such families has been found to be influenced by their interactions with mental health professionals. Families that have ongoing contact with the mental health professionals are more likely to recover from the crisis and cope with the situation. Home based treatment programs for children and adolescents with mental disorders appear to be an effective and sustainable strategy for meeting mental health needs in this group [2]. These programs would also be cost effective in countries where the health system is overburdened with infectious diseases and where psychiatric inpatient-care is limited. However, the success of such interventions requires compliance of patients and parents, and support from highly skilled therapists.

Parents and guardians play a major role in helping children grow and develop to their full potential. As children grow in the families they most significantly depend on their parents or guardians for basic needs support such as food, shelter, education, protection and care at all times but especially during life difficulties and times of crisis. Mental disorders in childhood and adolescence can be chronic and very disturbing, requiring proper attention, help and support from caregivers [3]. Thus, parents or guardians and relatives living with children with mental illness have additional responsibilities and roles to care for them as they do for other healthy children. In this study ‘children’ means any male or female persons not more than twelve years of age, and a ‘parent’ is a biological mother or father or anybody who assumes that role. The importance of family support for the growth and development of children and the role it plays as a determinant of whether children will receive mental health care or not, can not be overemphasized [4].

From the past until now the World Health Organization (WHO) mental health programme has not given due weight to child and adolescent psychiatry as compared to adults and the elderly [3]. Yet from a demographic and epidemiologic point of view, mental disorders in children and adolescents represent an important area that needs proper attention. It is estimated that up to 20 % of children and adolescents suffer from debilitating mental illness [5].

There are various ways in which child and adolescent mental disorders can be considered. One way is looking at these disorders in a priority manner based on their frequency of occurrence, degree of impact, therapeutic possibilities, and long term care effects [3]. From this perspective, child and adolescent mental disorders that pose a significant concern include learning disorders, hyperkinetic disorders (ADHD), depression and its associated suicide. Others include psychosis, pervasive development disorders, attachment disorders, anxiety disorders, conduct disorder, substance abuse and eating disorders.

Specialized mental health services for children have not yet been established in Tanzania [6]. The few existing mental health facilities in the country are mainly for adults. Children with mental disorders are treated in general wards and alongside adult mental patients. Psychiatric patients (including children and adolescents) are exempt from cost sharing charges for treatment. Medication is available but not always due to limited government support. When not available, parents or guardians are supposed to buy medicine for their children from private pharmacies. Moreover, the country lacks health care workers who are specialized in child and adolescent mental health. There are very few special schools for children with disabilities including those with mental disorders. Initiatives to advocate for social welfare of children with disabilities are taking place and the government is aware of that.

The major aim of this study was to explore challenges parents or guardians experienced while caring for their mentally ill children. Specifically, it aimed at identifying the psychological, emotional, and social problems they faced by living with a mentally ill child in the family, ways in which child mental illness interfered with economic activities in the family, and determining ways through which parents or guardians addressed these challenges.

Parents and guardians as main family care takers play a vital role in caring for mentally ill individuals including children and adolescents. Learning the challenges they face in caring for children and adolescents with mental illness is the first step in identifying ways to improve support for such caregivers. It is crucial that children receive appropriate care and support at home and during the outpatient visit to the hospital in order to meet their mental health needs. The scarcity of published studies in this area in Tanzania underscores the importance of this study to contribute towards better understanding of challenges faced when caring for mentally ill children. This study reports the psychological and emotional, social, and economic challenges parents and caregivers experienced and their reaction to the situation.

## Methods

The study was conducted at the Psychiatric Unit of Muhimbili National Hospital (MNH) in Dar es Salaam, the economic centre and fastest growing city in Tanzania with a population of more than 3 million people. It has three districts namely Ilala, Kinondoni and Temeke. MNH is located in Ilala and is the nation government referral hospital with the highest specialized health services. It is a place where patients with complicated health problems from all over the country can be found. The psychiatric unit is one of the departments in the directorate of clinical services at MNH. Unlike other departments which receive referrals from all over the country, this unit only caters for patients who are referred from the three districts. Psychiatric services at this unit are provided in two major forms depending on age. Firstly, it is provided for adults who receive either inpatient or outpatient care, and more serious patients who require long term hospitalization are referred to Mirembe hospital located in Dodoma region which is a specialized national psychiatric hospital. Secondly, there is child and adolescent care which is usually provided on the outpatient basis. A special day has been allocated every week (Thursday) for children and adolescents to be seen by health care providers. According to the records obtained at the unit, about 30 children and adolescents were seen every week.

Respondents were parents or guardians who brought their children to attend the weekly outpatient psychiatric clinic at the unit. Convenience and purposive sampling methods was used. Respondents were recruited through the identified child and adolescent nurse counselor working at the psychiatric unit. The first author worked together with the counselor in recruiting respondents while waiting for their children to be seen by the health care providers. The inclusion criterion was a parent or guardian who had lived for at least six months with the mentally ill child. This was considered an adequate period for having reasonable experience. Parents/guardians who met this criterion and consented were included in the study.

Focus group discussions (FGDs) and in-depth interviews were used to gather data. A semi-structured interview guide was used to interview respondents. All interviews were audio-recorded and a note book was used to take field notes. During FGDs the moderator (author) led the discussion and kept the conversation flowing while the research assistant was recording the interviews and taking field notes. Basic demographic data was also collected from respondents after conducting the interviews. All interviews were transcribed verbatim. Two FGDs and 8 in-depth interviews were conducted. The first FGD was attended by 5 respondents (all were mothers) and the second one by 6 respondents (3 fathers and 3 mothers). Eight in-depth interviews were conducted with 7 biological mothers and one grandmother. Conducting in-depth interviews was stopped when no more information could be obtained from caregivers. The purpose of using FGDs was to help get general information and ideas that were further explored during in-depth interviews.

Analysis of data was done by using content analysis which consisted of reading and re-reading the text, manual coding in the margins, and through memos, synthesizing and grouping of data in relatively exhaustive categories [7]. Data was analyzed in the original language (Swahili) in order to minimize the possibility of losing the original meaning of concepts. Analysis of data was done by the two authors and whenever there was a discrepancy in forming codes, categories or themes discussions were done to reach a consensus. Additionally, the analysis process was audited by a third person who was not part of the study but who is conversant with qualitative methods. Translation into English was done for what was included in the report.

The ethical approval of the study was obtained from the Research and Publications Committee of Muhimbili University of Health and Allied Sciences and permission to conduct the study was obtained from Muhimbili National Hospital. Written informed consent was sought from all participants prior to interview sessions.

## Results

The respondents’ demographic data and their children’s particulars are summarized in Table 1.

**Table 1:** Caregivers’ demographic data and children’s particulars

**ID. NO**	**SEX**	**AGE**	**MARITAL STATUS**	**TYPE OF CAREGIVER**	**LEVEL OF EDUCATION**	**OCCUPATION**	**CHILD’S AGE (YRS) /SEX**	**CHILD’S DIAGNOSIS**
ID10501JAii F10424JAi	F	33	Married	Mother	STD VII	Home Mother	10/F	Autism
F10424JAii	F	37	Married	Mother	STD VII	Home Mother	11	Mental retardation
ID10501JAi F10424JAiii	F	33	Widow	Mother	STD VII	Home Mother	7/F	Seizure disorder & ADHD
ID10430JAi F10424JAiv	F	36	Single	Mother	STD VII	Petty Business	9/F	Seizure disorder & Mental retardation
ID10430JAii F10424JAv	F	60	Married	Mother	None	Home Mother	8/M	Autism & Seizure disorder
ID10502JA	F	51	Widow	Grandmother	STD VII	Home Mother	3/F	Seizure disorder & Mental retardation
ID10503JA	F	33	Married	Mother	None	Petty Business	10/F	Epilepsy & Learning disability
ID10504JA	F	39	Married	Mother	Form IV	Catering and Decoration	11/F	Autism & Seizure disorder
ID10506JA	F	42	Married	Mother	Bachelor’s Degree	Police Officer	5/M	ADHD & Seizure disorder
F10508JAi	M	38	Separated	Father	STD VII	Vehicle mechanic	11/M	ADHD & Mental retardation
F10508JAii	F	26	Widow	Mother	None	Home Mother	8/M	Seizure disorder & Mental retardation
F10508JAiii	M	38	Married	Father	Bachelor’s Degree	Engineer	5/M	Autism
F10508JAiv	F	37	Married	Mother	STD VII	Home Mother	12/M	Epilepsy & mental retardation
F10508JAv	M	45	Married	Father	STD VII	Petty Business	9/F	Mental retardation & Epilepsy
F10508JAvi	F	40	Married	Mother	STD VII	Home Mother	11/M	Autism & Seizure disorder

NB: STD means standard (used to indicate the level of primary education e.g. STD VII means primary education level 7, which is the highest level of primary education in Tanzania).

### Psychological and emotional challenges

Four major themes emerged from the study that explain the psychological and emotional challenges that parents experience in the everyday life of caring for the mentally ill child. These were: disturbing thoughts, emotional disturbance, unavoidable situation, and communication problems.

A number of respondents revealed having disturbing thoughts about living with a mentally ill child. They expressed being stressed by the explicit behaviour of the child that caused problems not only for the parent but also to people nearby such as neighbours. Behaviours of the children that were of particular concern to parents were being aggressive, destructive, restless or hyperactive, making noise, and lack of proper eating skills. A father of a child with autistic disorder explained:

"“It is very true, it’s a problem, there is a problem because the way he is you can clearly see that he completely does not fit in the community. His actions are different and, of course, not accepted by other people. You may decide to go with him to some place, for example if you look at the appearance of my own child you may think he is just okay. But his actions are so disgusting (kukera) that you can’t go with him anywhere; to church or so, he just has to remain at home, it’s really a problem”. {F10508JAiii}"

Worrying about the future life of the child was another form of disturbing thought that some parents experienced due to the inability of the child to accomplish personal and social needs such as self-care and education. They showed these concerns when the child could not do certain developmental tasks expected at specific ages such as feeding, toileting, bathing, and dressing, as expressed by a mother of a child with autistic disorder:

"“Truly, what I am afraid of is that it will be more difficult later in life. This is what makes me fail to sleep all the days, I keep thinking only about that and my husband and I quarrel about that everyday. Just imagine, now you have to wash her since she soils herself with faeces and urine, now when she grows up it will become a very big task, it will really become a big task. … My greatest concern is after menarche (kuvunja ungo); what I am thinking of is after menarche and this is a big test”. {ID10501JAi}"

Parents were also disturbed by the complexity of caring responsibilities that demanded a lot of work and being available most of time to meet the daily needs of the child. The issues about how to handle the child and ensure security if the parent died was expressed by one father of a child with mental retardation and epilepsy:

"“I accept to be meek and gentle because I know it is God who has intended for the matter to be like this, but it’s a big burden to me because that child is always restless. He can not even sit down with others for a minute, he likes seeing himself wandering and he feels good. So in the process of wandering he gets lost, and there we are staying near the road. Therefore the mother doesn’t do anything other than looking after (kumwangalia) the child all the days of her life for two years now, and we don’t know what will happen in the future.” {F10508JAv}"

A mother of a child with ADHD and seizure disorder said,

"“Until now I don’t know how my child’s condition will be if, let’s say I die. This is because I don’t have any sister, mother, or father who can take care of my child in that condition ………let’s say I die now, I don’t think she will be in a better condition…”{ID10501JAi}"

Living with a mentally ill child was described as emotionally distressful by parents. Varying degrees of emotional distress were experienced which included having feelings of sadness and inner pain or bitterness. Parents experienced these negative emotions due to disturbing behaviour of the children, extra care-giving responsibilities, family and social problems caused by the child, and people’s perception about families having a mentally ill child. A sad mother of a child with autism explained:

"“I really somehow grieve (sononeka), I just accepted it (having a mentally ill child), but I really grieve because I really suffer (pata shida) a lot from this child…now when I look at this child with disability and the challenges of care, it makes me grieve…” {ID410501JAii}"

Parents described living with or having a mentally ill child as a disturbing and yet unavoidable situation. They had to accept it since they had no other alternative. They viewed their caring duty for the child as very difficult and distressing. This was stated by some participants when they were responding to the question about how they generally viewed the situation of having a mentally ill child at home. One mother of a child with autistic disorder said,

"“Really I have accepted it because I have already been given, but it’s a big task, it’s a very big task to care for a child with mental disability”. {F10424JAi}"

Another mother of a child with mental retardation and seizure disorder emphasized:

"“…yes we have been disgusted (tunakerwa), but God has already planned it for you, and you have no any other way”. {F10508JAii}"

The inability of the child to express needs was another source of psychological and emotional distress associated with living with mentally ill children. This caused parents to be unable to understand the child especially when he/she had problems. Sometimes when the child went out, he/she was mistreated by other people and returned home crying. When he/she was asked by the parent to explain what happened he/she couldn’t explain, as expressed by a father of a child with autism:

"“The challenge I face is that that child can not express his needs … Sometimes he may be sick and you don’t know, he just cries, when he has problems he just ends up crying. Now you don’t know why he is crying, and sometimes when you touch him and feel that he is hot then you may guess that this could be malaria and take him to hospital. But when he has like stomach ache you can’t understand, you just see him crying,…” {F10508JAiii}"

### Social challenges

Caring for a child with mental illness was found to be associated with many social challenges. Social services, stigma and caring responsibilities were areas which posed major concerns for parents. Other important issues included a lack of public awareness, social support, and social life.

Inadequate social services for children with mental disorders was the most challenging issue for parents. They were concerned about education for the child and to a lesser degree hospital care. The most distressing aspect was inability of the child to acquire education due to an inadequate number of schools. Parents spent a lot of time looking for schools that could accommodate the child as explained by a mother of a child with mental retardation and seizure disorder:

"“…I was told to take her to Uhuru Mchanganyiko (primary school for children with various disabilities); I went there because she has mental and visual problems. When I got there they told me they would not accept the child and that I should take her to Mtoni Special School (another school for disabled children). Until now, when I go to Mtoni special school they tell me there is no vacancy. There are no school opportunities for these children or their schools are very few, you see.” {F10424JAiv}"

Parents whose children were lucky to be recruited in the special schools were experiencing other problems. Their children could not understand anything that was taught at school and had transport problems especially because of the traffic congestion in the city of Dar es Salaam. They could not take their children to school because they could not afford bus fare, as explained by the mother of a child with autism and seizure disorder:

"“She goes to school but she doesn’t understand…… she just goes to school but there is nothing she understands at school” {ID10504JA}"

Another mother of a child with autism and seizure disorder added:

"“The first difficult thing about it is that I haven’t found the school. Money is needed, as he (the child) is supposed to stay there (at school) because we can not afford for him going and coming back since we are not able to. Schools like Buguruni require having money.” {ID10430JAii}"

With regard to health care services, parents were generally satisfied with services at the MNH Psychiatric Unit, as expressed by the mother of a child with epilepsy and learning disability:

"“The service she (the child) receives is really good, they do their best at the hospital…I am satisfied with this service.” {ID10503JA}"

However parents complained about: long waiting periods before they were seen by the doctor, spending too little time with the doctor, out of stock medications, lack of speech therapists in the country, and lack of proper facilities such as toilets. A mother of a child with seizure disorder and mental retardation complained:

"“With hospital services, for example it is very far where I come from, you can arrive there (at the clinic) maybe at 8:00 AM. From that time you may see the doctor may be at 11:00 AM. So it really becomes a problem because you sometimes leave home before the child had tea. Now you stay there with the child until 11:00 AM, it becomes a problem…I would like that if we just get there we should be seen early, then we leave. Sometimes you may stay there for a long time and because of hunger the child starts troubling you, it really is a problem. … I was not happy with that thing, the toilet, it should have been a squat toilet (choo cha chini) (as opposed to western type) the normal one, that would have been good”. {ID10430JAi}"

Living with mentally ill children was found to be associated with stigma. Parents were troubled by the mentally ill child being mistreated, discriminated against and segregated in the community. Sometimes the child was told words that made him/her feel bad. Parents were laughed at and told bad things about the child.

The child was mistreated by people in various ways. Sometimes he/she was labelled and made fun of at school, considered to be useless and even rejected by the parent because of the disability. A mother of a child with epilepsy and learning disorder explained:

"“…My child is in primary school, she goes to school and comes back complaining that she is being called a crazy person (tahira). She really feels bad (anajisikia vibaya) and when she comes to the hospital she tells her doctors…” {ID10503JA}"

Parents were sometimes held responsible for the child’s behaviour. They were thought to be spoiling the child by not being strict enough when the child was behaving strangely. Furthermore, some parents were told they had caused the child to become mentally ill as a means of getting rich and becoming successful in life (it is common to see disabled children with their parents in the streets begging). A father of a child with ADHD and mental retardation explained with sadness:

"“Let me add to what I have said. It is true, according to the prevailing situation when people see those children the majority of them say we have caused them to become sick as way of getting money and becoming successful in life.” {F10508JAi}"

The burden of caring for the mentally ill child was found to be mainly borne by the mother. Mothers complained about the role that fathers played in everyday care of the child. They expressed that some fathers were only supporting the child financially and others did not care at all. The grandmother who was taking care of the child with seizure disorder and mental retardation whose mother was dead commented:

"“…Now the burden of care is upon me. The father doesn’t love her (the child) very much. Sometimes you may tell him that you have a problem with the child and he will tell you to just wait. Now that’s just like totally not being involved in the care of the child.” {ID10502JA}"

Ignorance about mental disorders was perceived by caregivers to be common in the community. Some children with mental illness and their parents suffered stigma and mistreatment out of ignorance. A mother of a child with autism and seizure disorder explained:

"“The public should understand these children. If you go with her to some place everybody is surprised, you know, even the child wonders why they get surprised at her. They really don’t understand and I don’t know how. They think it’s something that does not exist…” {ID10504JA}"

Parents in this study expressed their concern about not receiving the needed support from neighbours and people in the community. Some people could not even give help when they found the child in a critical condition like having seizures. One mother of a child with autism complained that people sitting in the commuter bus would not help her by giving her a seat when she was standing with the child on her back.

"“I have to carry the child from home, board the bus …somebody in the bus may see you carrying the child while you are standing without even letting you sit.” {F10424JAi}"

The social life of parents was found to be disrupted by the presence of a mentally ill child in the family. Sometimes parents avoided going with the child to social gatherings such as church because of the child’s disturbing behaviour. Parents also experienced conflicts in the community and sometimes were even accused when the child destroyed somebody’s property. This created tension and resulted in lack of peace especially with people like neighbours. Sometimes the love life of a mother was affected for reasons related to having a mentally ill child. One mother of a child with ADHD and seizure disorder who avoided having another child with a new partner in case she was overwhelmed by caring responsibilities explained:

"“…Who knows? He (the partner) went to see his friends there… they said to him, “How can you stay with a woman and just take care of her child? After all she (the child) is crazy, and so on. She doesn’t want to have a child with you, I don’t know what!” Then that person (the partner) ran away from me, we were staying in the same room.” {ID10502JA}"

It was noted that mentally ill children were vulnerable and suffered mistreatment from people by being beaten, pushed, and burned. The child who could not speak seemed to be more vulnerable since he/she could not mention a person who was responsible for the cruel action when parents wanted to know. A mother of a child with ADHD and seizure disorder said,

"“…may be she (the child) goes and touches somebody’s property, or in doing so she makes somebody’s water dirty. The owner will come out with anger and will beat the child with a fist in the head or pinch her knowing that the child will not say. The child comes back crying and when you ask her she doesn’t understand you.” {F10424JAiii}"

These children were also reported to be at risk of being physically and sexually abused such as being burned or raped as explained by a mother of a child with seizure disorder and mental retardation:

"“…for example, one day I left my child with the house girl. She dared to take the spoon that she was using for frying and applied it on the child’s skin. When I came back I found the child with marks of wounds on the body………there is another child in the neighbourhood who is also disabled. That day her mother went to the field in the morning leaving the child still asleep with instructions to follow her to the field. When that child got out to follow her mother there was a man nearby who called her into his house and did to her a very bad thing (shedding tears), just last week…he raped her. We really need to be very close to these children. The issue of being raped makes us to be with them so that they don’t suffer such actions” {F10424JAiv}"

### Economic challenges

Three major themes emerged that explained how living with a mentally ill child interfered with economic activities of the families. These were: existing poverty, interference with various income generating activities, and extra expenditure due to the illness.

Poverty was revealed by parents as being responsible for their inability to meet certain important needs of the mentally ill child. Some parents were house wives who did not have any means of earning income and depended solely on their partners; those who did not have partners expected to get help from other people, especially relatives. This was a problem if they could not get the help they needed. They (including fathers) could not manage buying drugs for their children when they did not receive them at the hospital. They also could not afford bus fare to attend the clinic with their children on the day of their appointment. A mother of a child with autism explained:

"“…From here you may go to the hospital’s drug unit…you may find that you get only one type of drugs while you have a prescription of two or three drugs… If you go to a (private) pharmacy you find that it’s 500 shillings (US$ 0.36) per pill. Now with our income in this situation; house rent, water bills, everything, transport charges, you may reach a point where – I once spoke with my mouth, and may God forgive me, that instead of giving me these problems he should have taken him (the child), I reached that point…” {F10508JAi}"

Daily life and activities of parents in this study were very much affected by the presence a mentally ill child. Much time was spent looking after the child and as a result they were not able to do other important activities such as business. Income generation in the family was affected and this further escalated family poverty as explained by a mother of a child with seizure disorder and mental retardation:

"“Care responsibilities for such children are very cumbersome; you need to stay with them for a very long time so that you watch over them from morning till evening. Your activities will be limited only to the home environment; you can’t go out for activities to earn a living apart from being home.” {F10424JAiv}"

### Measures to address challenges

Parents expressed various ways they used in order to address the challenges they were facing by living with a mentally ill child. A variety of coping mechanisms were employed in different situations depending on what seemed to be helpful to the parents. They sought professional and spiritual help (from religion and traditional healers). One mother whose child is afflicted with autism and seizure disorder expressed:

"“It is the hospital, it’s the hospital that has helped her (the child) to be honest. If it was not for the hospital her condition would have been worse. I have done everything I could; I have not stopped going to the hospital since she begun having the problem. It is 11 years now she is on medication…” {ID10504JA}"

Other measures parents took included training the child to do what she/he could not do such as toilet training and speaking, involving other family members such as siblings in the care of the child, and seeking information about the child’s disorder from sources like the internet. Due to the nature of some disorders and the behaviour displayed by the child, some caregivers tried to control the child’s environment for safety reasons.

The focus group discussions and in-depth interviews served as psychological relief for parents as they had the opportunity for somebody to listen to them. After a FGD one respondent who had a child with autism gave a comment which was supported by the rest:

"“Just having somebody listen to you like this makes you feel better”. {FN10508JA}"

This was also the case at the end of an in-depth interview with another respondent whose child was afflicted with ADHD and seizure disorder:

"“Thank you very much Mr. Joel (the author), I am also happy because I have at least had someone to listen to me, I have never had such opportunity…”. {ID10506JA}"

## Discussion

The findings of this study revealed various psychological, social and economic challenges that parents experienced in living with a mentally ill child. Some of the key themes found in the peer review literature with respect to experiences and needs of families of individuals with mental illness [8] also emerged in this study.

### Psychological and emotional challenges

The psychological and emotional challenges experienced by parents in this study are similar to what was found in the United States [9] where mothers of children with serious mental illness had concerns about the future of their children due to the child’s special needs, erratic or worsening of behaviour, and long term consequences. Similar stressful experiences were also reported by parents of children with autism [10] and intellectual disability [11,12]. Emotional experience of sadness, and inner pain or bitterness associated with having a mentally ill child differs from a previous study in the United States [9], where emotional strain was expressed by mothers as having fears, frustrations, and guilt in dealing with the child’s behaviour and attitude. Parents had similar communication problem with their children as in the United States and United Kingdom [13,14]. Lack of speech and language therapists is a problem experienced not only in a low income country, but in high income countries as well.

Providing psychological and emotional support for caregivers of mentally ill children should be considered to give them some relief from distress. One of the ways to achieve this is creating the environment for health professionals to be working closely with caregivers to effectively treat their children’s mental illness [15]. Provision of information through booklets on how to manage the child can be helpful [12]. Nurses can also play a role in providing social and emotional support [9]. Working closely with mental health professionals facilitates recovery of families with mentally ill children from crises and coping with the situation [1].

### Social challenges

Challenges of inadequate social services experienced by parents in this study are similar to research conducted in the United Kingdom [14] but different from another study [10] where parents reported high proportion of their autistic children living in residential provisions including weekly boarding at the school. This entails the need for the government, private sector, and non-governmental organizations to consider school expansion programs for people with disabilities such as children with mental disorders.

Parents in this study were actually advocating for boarding schools for their children, though this contradicts with the existing evidence that home based treatment program for children and adolescents with mental disorders appears to be an effective and sustainable strategy for meeting their mental health needs [2]. Since the success of home based care interventions requires compliance of patient and parents, and support from highly skilled therapists [2], provision of professional support to caregivers could alleviate the burden of care. Respite services, which no parent reported receiving, might be helpful in these cases since it is one of the perceived needs of families in dealing with challenging behaviour of children [16]. Other interventions such as establishing day care centres for such children could bring relief to caregivers as it would save much time spent to look after them and in turn this precious time could be used for other productive activities. Moreover, systems such as hospitals and schools need to connect in evidence based practice for effective interventions such as reaching children in their natural settings, designing interventions that fit into these contexts, and working with families and local communities [4]. Special education for such children is very important as far as parents in this study were concerned.

Stigma experienced in this study has also been reported in other settings [3,17,18]. Public awareness programs about children with mental disorders at all levels of society is necessary in order to reduce stigma. Structure of mental health services need to be improved to reduce issues of dissatisfaction by clients. More speech therapists need to be trained and made available.

The vulnerability of mentally ill children for being physically and sexually abused is an important issue that needs to be well addressed. Tanzania ratified the Convention on the Rights of the Child in 1991 and since then children have survival rights, development rights, protection rights, participation rights, and the right not to be discriminated against. However these rights have often been violated by the community, parents, and guardians. Particularly, deficiencies in enforcement of the laws concerning children have contributed to denial of these rights. [19]. Although some legal actions against perpetuators of child abuse were reported in this study, more efforts are needed for the government and local communities to protect these children. Enforcement of laws that protect children need to be strengthened [20]. Measures need to be taken to promote the rights of the child such as mobilizing the community against harmful traditional practices and making parents accountable for caring for their children [19]. All these measures are in accordance with the Universal Declaration of Human Rights [21].

### Economic challenges

The economic challenges that caregivers experienced in this study were mainly due to poverty, child care interfering with various activities such as business, and extra expenses associated with the illness. Similar poverty challenges were experienced by families of children with intellectual disabilitties in United Kingdom [22] when they were compared to families without such a child. Child care interfering with various activities corresponds with a study in Australia [23] where caregivers experienced disruption to achieve their own goals/dreams. Challenges due to poverty could be addressed by providing financial assistance, food and clothing [24]. But the best way to help these caregivers may be to facilitate acquisition of adequate time for them to do their own income generating activities. This can be achieved by providing respite services and establishing day care centres for their ill children so that they do not spent so much time looking after them, and by providing loans and perhaps training them to start some form of business. These are actually some of the ideas they suggested during the interviews, and the government, private sector, and NGOs could investigate the possibility of providing such economic support.

### Measures to address challenges

Parents took various measures in order to deal with challenges they faced in living with the mentally ill child. They sought professional assistance from the hospital, spiritual help from their religious leaders and traditional healers, and involved other family members in child care. They also trained the child to do self-care, sought information from the internet, received advice from experienced parents, and tried to control the child’s environment. These measures correspond with some accommodation variables of the eco-cultural model [25] which explains how families respond and cope with having a member with chronic disability.

### Limitations of the study

This study explains experiences of caregivers in the given setting and nature of disorders suffered by their children. It is at the reader’s discretion to see how the results can be applied in other similar settings and circumstances.

## Conclusion

Caregivers of children with mental disorders experience many psychological, social, and economic challenges. These include stress, worries, sadness, grief, bitterness, inadequate special schools for their ill children, stigma, lack of social support, disruption in social life and poverty. Professional assistance, public awareness of mental illnesses in children, social and financial support by the government, private sector, and NGOs are important in addressing these challenges.

## **Abbreviations**

ADHD, Attention Deficit Hyperactive Disorder; DSM-IV-TR, Diagnostic and Statistical Manual of Mental Disorders, 4th Edition, Text Revision. (A classification system of mental disorders published by the American Psychiatric Association {APA} that includes all currently recognized mental health disorders.); FGDs, Focus group discussions; IEC, Information, Education, and Communication; MNH, Muhimbili National Hospital; NGOs, Non Governmental Organizations; WHO, World Health Organization; ID10430JAi/ F10508JAi, Represents a quote from the participants; , ID stands for in-depth interview and F for focus group discussion. The following two digits in each case stand for the year of interview, the next one digit is for the month and the other next two digits for the date. The following capital letters (JA) stand for initials of the name of the moderator and the last small letters (i or ii), if any, stands for whether it was the first or second interview/discussion done on that same day.; , Example: ID10430JAi means in-depth interview conducted in the year 2010 on April 30th. The moderator was Joel Ambikile and it was the first in-depth interview on that day.

## Competing interests

The authors declare that they have no competing interests.

## Authors’ contributions

JSA did all the work from research proposal development, data collection and analysis, and report writing. Dr. AO supervised the whole work from proposal development, ensuring proper data collection instruments, relevant data analysis method, and adherence to ethical issues and paper writing skills. She was involved in the step by step process of data analysis and formation of themes.

## References

[B1] Helena G Gun P Bengt F Mental health professional support in families with a member suffering from severe mental illness: a grounded theory model Scand J Caring Sci 2006 20 1 102 109 10.1111/j.1471-6712.2006.00380.x 16489966

[B2] Schmidt MH Lay B Gopel C Naab S Blanz B Home treatment for children and adolescents with psychiatric disorders Eur Child Adolesc Psychiatry 2006 Aug 15 5 265 276 10.1007/s00787-006-0531-x 16604436

[B3] WHO Caring for children and adolescents with mental disorders Setting WHO directions 2003 World Health Organization, Geneva

[B4] Kazak AE Hoagwood K Weisz JR Hood K Kratochwill TR Vargas LA A meta-systems approach to evidence-based practice for children and adolescents Am Psychol 2010 65 2 85 97 20141264 10.1037/a0017784

[B5] WHO The World Health Report 2000 Health systems: Improving performance 2000 The World Health Organization, Geneva

[B6] WHO Mental Health Atlas 2005 United Republic of Tanzania 2005 World Health Organization, Geneva

[B7] Graneheim UH Lundman B Qualitative content analysis in nursing research: Concepts, procedures and measures to achieve trustworthiness Nurse Educ Today 2004 24 2 105 112 10.1016/j.nedt.2003.10.001 14769454

[B8] Riebschleger J Scheid J Luz C Mickus M Liszewski C Eaton M How are the experiences and needs of families of individuals with mental illness reflected in medical education guidelines? Acad Psychiatry 2008 32 2 119 126 10.1176/appi.ap.32.2.119 18349331

[B9] Scharer K Colon E Moneyham L Hussey J Tavakoli A Shugart M A comparison of two types of social support for mothers of mentally ill children J Child Adolesc Psychiatr Nurs 2009 22 2 86 98 10.1111/j.1744-6171.2009.00177.x 19490279 PMC2727132

[B10] Hastings RP Child behaviour problems and partner mental health as correlates of stress in mothers and fathers of children with autism J Intellect Disabil Res 2003 47 4–5 231 237 12787155 10.1046/j.1365-2788.2003.00485.x

[B11] Duvdevany I Abboud S Stress, social support and well-being of Arab mothers of children with intellectual disability who are served by welfare services in northern Israel J Intellect Disabil Res 2003 47 4–5 264 272 12787158 10.1046/j.1365-2788.2003.00488.x

[B12] Hudson AM Matthews JM Gavidia-Payne ST Cameron CA Mildon RL Radler GA Evaluation of an intervention system for parents of children with intellectual disability and challenging behaviour J Intellect Disabil Res 2003 47 Pt 4–5) 238 249 12787156 10.1046/j.1365-2788.2003.00486.x

[B13] Little L Clark RR Wonders and worries of parenting a child with Asperger syndrome & nonverbal learning disorder MCN American Journal of Maternal Child Nursing 2006 31 1 39 44 10.1097/00005721-200601000-00009 16371824

[B14] Wodehouse G McGill P Support for family carers of children and young people with developmental disabilities and challenging behaviour: what stops it being helpful? J Intellect Disabil Res 2009 Jul 53 7 644 653 10.1111/j.1365-2788.2009.01163.x 19298502

[B15] Fabiano GA Pelham WE Evidence-based treatment for mental disorders in children and adolescents Curr Psychiatry Rep 2002 Apr 4 2 93 100 10.1007/s11920-002-0041-6 11914169

[B16] Turnbull AP Ruef M Family perspectives on problem behavior Ment Retard 1996 Oct 34 5 280 293 8908993

[B17] Hinshaw SP The stigmatization of mental illness in children and parents: developmental issues, family concerns, and research needs Journal of Child Psychology and Psychiatry 2005 Jul 46 7 714 734 10.1111/j.1469-7610.2005.01456.x 15972067

[B18] Larson JE Corrigan P The stigma of families with mental illness Acad Psychiatry 2008 32 2 87 91 10.1176/appi.ap.32.2.87 18349326

[B19] Government Child Development Policy Ministry of Community Development WAaC, ed.: Government of the United Republic of Tanzania 1996

[B20] OAU African Charter on the Rights and Welfare of the Child Unity OoA, ed 1990

[B21] UN Nations U Universal Declaration of Human Rights United Nations High Commissioner for Human Rights 2006

[B22] Emerson E Mothers of children and adolescents with intellectual disability: social and economic situation, mental health status, and the self-assessed social and psychological impact of the child's difficulties J Intellect Disabil Res 2003 47 4–5 385 399 12787168 10.1046/j.1365-2788.2003.00498.x

[B23] Wynaden D The experience of caring for a person with a mental illness: A grounded theory study Int J Ment Health Nurs 2007 16 6 381 389 10.1111/j.1447-0349.2007.00493.x 17995509

[B24] Nyati Z Sebit MB Burden of mental illness on family members, caregivers and the community East Afr Med J 2002 79 4 10.4314/eamj.v79i4.8880 12625678

[B25] Intellectualdisability http://www.intellectualdisability.info/families/P_family_issues_rj.html Family Issues. 2003 [cited 2009 12/05]; Available from:

